# Long-Term Clinical Profile of Children With the Low-Penetrance R92Q Mutation of the *TNFRSF1A* Gene

**DOI:** 10.1002/art.30237

**Published:** 2011-04

**Authors:** M A Pelagatti, A Meini, R Caorsi, M Cattalini, S Federici, F Zulian, G Calcagno, A Tommasini, G Bossi, M P Sormani, F Caroli, A Plebani, I Ceccherini, A Martini, M Gattorno

**Affiliations:** 1 Istituto G. GasliniGenoa, Italy; 2 Spedali Civili and University of BresciaBrescia, Italy; 3 University of PaduaPadua, Italy; 4 AOU G. MartinoMessina, Italy; 5 IRCCS Burlo Garofolo and University of TriesteTrieste, Italy; 6 IRCCS Policlinico S. MatteoPavia, Italy; 7 University of GenoaGenoa, Italy

## Abstract

**Objective:**

To analyze the long-term impact of the R92Q mutation of *TNFRSF1A* in children with periodic fever, in comparison with children with tumor necrosis factor receptor–associated periodic syndrome (TRAPS) with *TNFRSF1A* structural mutations and children with periodic fever of unknown origin fulfilling the criteria for periodic fever, aphthosis, pharyngitis, and adenitis syndrome (PFAPA).

**Methods:**

The extracellular region of *TNFRSF1A* was analyzed in 720 consecutive children with periodic fever, using denaturing high-performance liquid chromatography and DNA sequencing. Followup data on 11 pediatric patients with *TNFRSF1A* structural mutations (cysteine or T50M), 23 pediatric patients with an R92Q substitution, and 64 pediatric patients with PFAPA were collected during routine clinic visits. The 50-item Child Health Questionnaire was used to assess health-related quality of life (HRQOL).

**Results:**

The frequency of typical TRAPS-related clinical manifestations was significantly lower and the impact of the disease on HRQOL was significantly reduced in patients with the R92Q mutation compared with TRAPS patients carrying structural mutations of *TNFRSF1A*. Followup data on 11 TRAPS patients with *TNFRSF1A* structural mutations (mean followup 7.9 years), 16 patients with theR92Q substitution (mean followup 7.3 years), and 64 patients with PFAPA (mean followup 5.2 years) were available. Patients with R92Q mutations and patients with PFAPA displayed a higher rate of self-resolution or amelioration of the fever episodes than did TRAPS patients with structural mutations.

**Conclusion:**

Although some cases may progress to a more chronic disease course, the majority of children with an R92Q mutation of the *TNFRSFA1* gene show a milder disease course than that in children with *TNFRSFA1* structural mutations and have a high rate of spontaneous resolution and amelioration of the recurrent fever episodes.

Tumor necrosis factor receptor–associated periodic syndrome (TRAPS) is an autosomal-dominant disease caused by mutations of the type I tumor necrosis factor receptor (TNFRI) gene (*TNFRSF1A*) (1,2). The *TNFRSF1A* mutations reported in TRAPS patients include missense substitutions, mainly affecting the highly conserved cysteine residues of the extracellular cysteine-rich domains involved in disulfide bond formation and in the folding of the extracellular portion of TNFRI (3,4). These genetic variants (also defined as structural mutations) have a high penetrance, and the corresponding phenotype is characterized by a severe disease course. Patients display long-lasting fever episodes (duration of 1–3 weeks) associated with rash, arthralgia, myalgia, and abdominal pain. Progression toward a more chronic disease course and renal amyloidosis is a possible long-term complication in adulthood (5). Patients may require prolonged treatment with steroids and use of second-line drugs (3–5).

Among Caucasian populations, the R92Q mutation is the most frequently observed variant of the *TNFRSF1A* gene in children with periodic fever (6,7). R92Q is a missense and low-penetrance mutation with no relevant impact on the structure and function of the mutated protein and is usually associated with a milder disease course, characterized by episodes of fever lasting only a few days, lower intensity of disease-associated symptoms, and a much lower prevalence of amyloidosis (3,4). Notably, according to different studies, the allele frequency of the R92Q variant in the general population ranges from 1.2% to 4% (3,4,6,8). Apart from the description of very few anecdotal cases of renal amyloidosis in adults (9), no information is available on the long-term clinical profile of patients affected with TRAPS who are carriers of the R92Q mutation.

Indeed, only 10–20% of children with a clinical picture consistent with a periodic fever turn out to be carriers of at least one mutation of the genes known to be associated with monogenic periodic fevers such as familial Mediterranean fever, mevalonate kinase deficiency, and TRAPS (10). The clinical spectrum of the large group of mutation-negative children with periodic fever is extremely variable. In the pediatric population, the most common cause of periodic fever is a clinical entity characterized by recurrent episodes of fever, known under the acronym PFAPA (periodic fever, aphthosis, pharyngitis, and adenitis syndrome) (6,11,12). The genetic basis of this syndrome is not yet documented, and usually spontaneous resolution of the fever episodes will occur a few years after symptom onset. In the present study, we chose to analyze mutation-negative patients who fulfilled the PFAPA criteria, in order to have a more homogeneous disease control group.

The aim of the present study was to analyze the long-term clinical course in children with periodic fever carrying the R92Q mutation, as compared with that in TRAPS patients carrying structural mutations of *TNFRSF1A* and patients with periodic fever of unknown origin fulfilling the criteria for PFAPA.

## PATIENTS AND METHODS

### Patients

Starting from 2002, a nationwide laboratory facility for the genetic diagnosis of recurrent fevers in children was established at the G. Gaslini Institute (10). Up to July 2009, specimens from 720 consecutive patients with periodic fever were received from pediatric centers located all over Italy for molecular analysis of the *MVK*, *MEFV*, or *TNFRSF1A* genes. The extracellular region (from exon 1 to exon 6) of the *TNFRSF1A* gene was analyzed using denaturing high-performance liquid chromatography and DNA sequencing of amplimers displaying anomalous chromatographic patterns, as previously described (10).

Clinical data were collected at the time of molecular screening and at the last followup visit, using a standardized questionnaire (10). Informed consent was obtained from all subjects, with approval from the G. Gaslini Institute Ethics Board. The following variables were considered: family history (recurrent fever and/or manifestations of inflammation consistent with an autoinflammatory disease), number of fever episodes per year, mean duration of fever episodes, and presence and frequency (never, sometimes, often, always) of the clinical manifestations associated with episodes of fever.

A spontaneous disease course was classified according to the following 4 categories: 1) spontaneous resolution (no fever episodes during the last 6 months of followup, in the absence of any continuous steroid and/or second-line treatment); 2) spontaneous improvement (reduction of >30% in the number of fever episodes during the last 6 months of followup, in the absence of any continuous steroid and/or second-line treatment); 3) stable disease (reduction of ≤30% in the number of fever episodes, in the absence of any continuous steroid and/or second-line treatment; or 4) worsening of the disease (any increase in the frequency or duration of fever episodes or appearance of new major clinical manifestations [i.e., amyloidosis], in the absence of any continuous steroid and/or second-line treatment). In addition, the therapeutic strategy used during the disease course was investigated according to the following categories: 1) need for short courses of steroids administered on demand for not more than 5 consecutive days; 2) need for steroid courses administered on demand for a period longer than 5 days; or 3) need for a second-line treatment, due to steroid resistance or steroid dependency. Sixty-four pediatric patients who fulfilled the current criteria for PFAPA (12,13) and were negative for mutations of the *MVK, TNFRSF1A,* or *MEFV* gene were analyzed as disease controls.

### Evaluation of health-related quality of life (HRQOL)

The Italian national language version of the parent-administered 50-item Child Health Questionnaire (CHQ-PF50) (14) was used to assess the quality of life of the patients. The CHQ is a generic, self-administered instrument designed to assess the physical, emotional, and social components of health status in children. It includes 15 health concepts (score range 0–100) that explore both physical and psychosocial domains (15). Two summary measures based on the US normative standard, the physical summary score (PhS) and the psychosocial summary score (PsS), are provided; the summary measures are standardized to have a mean score of 50 and an SD of 10. Higher scores in the scales indicate better HRQOL. The CHQ-PF50 questionnaire was administered to all patients carrying *TNFRSF1A* gene mutations. The questionnaire was administered at the time of the first evaluation at the center. An international sample of 3,315 healthy children (52.2% of whom were female, mean ± SD age 11.2 ± 3.8 years) constituted the healthy control group, as previously described (16).

### Statistical analysis

Differences among groups were evaluated using the nonparametric Kruskal-Wallis test. Symptom frequency was evaluated using the chi-square test. Differences in HRQOL were analyzed with Student's *t*-test.

## RESULTS

### Genotype characterization

From 2002 onward, 34 pediatric patients carrying mutations of the *TNFRSFA1* gene were identified (Table 1). Eleven patients displayed mutations associated with a substantial modification of the structure of the protein (structural mutations) (3). Twenty-three patients carried the low-penetrance R92Q substitution in a heterozygous state. Two sisters carried the C88Y mutation (Table 1). Three patients with the R92Q mutation (2 boys and 1 girl) were siblings; the 2 brothers were heterozygous, whereas the sister was homozygous for the same variant (Table 1).

**Table 1:** Mutations of *TNFRSF1* and other genes involved in autoinflammatory syndromes in children with periodic fever

Mutation	No. of patients
Mutations of *TNFRSF1A*	
Structural	
T50M	3
C52Y	2
C55Y	2
C88Y	2
C29Y	1
C43R	1
Low-penetrance R92Q*	23
Concomitant mutations of other genes†	
*MVK* I268T/V377I	1
*MVK* H20Q/V377I	1
*MVK* T237S/T237S	1

* One of these patients carried the mutation in a homozygous state.

† Identified in patients with the R92Q substitution.

Nine (82%) of the 11 children carrying *TNFRSF1A* structural mutations had a family history consistent with an autoinflammatory disease (including renal amyloidosis in 3 families), for a total of 24 affected individuals. In each family, all of the affected members screened for *TNFRSF1A* carried the same mutation as that found in the proband. Complete penetrance was observed in all families harboring a structural mutation.

Apart from the family with 3 affected children, a positive family history of recurrent fever was noted in only 1 patient carrying the R92Q mutation. In this case, the mother reported experiencing recurrent episodes of fever during childhood and was positive for the R92Q mutation.

Asymptomatic parents of 12 patients carrying the R92Q mutation were also screened. In all cases, at least 1 parent carried the same variant as that in the proband. In the family of the 3 affected children with the R92Q variant, both parents carried the same mutation but were totally asymptomatic.

A positive family history of recurrent fever in childhood (mainly related to 1 parent only) was reported in 9 (14%) of 64 patients with PFAPA. Among children carrying the R92Q substitution, additional mutations co-occurring in other genes known to be associated with periodic fevers were found; 2 of these patients were compound heterozygous for the *MVK* gene (I268T/V377I in 1 patient and H20Q/V377I in the other patient) (17), while 1 patient was homozygous for the T237S variant of the *MVK* gene. These latter 3 patients were considered affected by mevalonate kinase deficiency, and therefore were excluded from the present study.

### Clinical and laboratory features of the patients at baseline

The main clinical findings observed from disease onset to the time of molecular analysis (baseline) were noted in 11 children carrying *TNFRSF1A* structural mutations (defined as TRAPS patients), 20 children with the R92Q substitution, and 64 patients with PFAPA, as shown in Table 2. Patients carrying structural mutations displayed prolonged episodes of fever, with a mean duration of fever of 15.3 days (range 3–18 days) compared with a mean of 5.9 days (range 3–15 days) in patients with the R92Q variant and a mean of 4.6 days (range 2–10 days) in patients with PFAPA. The mean number of attacks per year was 6.4 (range 1–12) in TRAPS patients with structural mutations, 10.3 in patients with the R92Q mutation (range 3–20), and 14.3 (range 4–20) in patients with PFAPA. At baseline, all patients displayed recurrent episodes of fever only, without other relevant symptoms between the flares.

**2:** Baseline demographic and clinical characteristics of the patients with TRAPS, patients with the R92Q mutation, and patients with PFAPA*

	Patients with TRAPS (n = 11)	Patients with R92Q (n = 20)	Patients with PFAPA (n = 64)	*P*†
No. male/no. female	6/5	11/9	38/26	NS
Age at disease onset, mean (range) years	3.1 (0.3–7.1)	3.6 (0.6–13)	1.6 (0.3–4.7)	0.005
Age at molecular analysis, mean (range) years	5.6 (1–9)	6.1 (1.2–15)	5.7 (1.5–15.1)	NS
Fever duration, mean (range) days	15.3 (3–18)	5.9 (3–15)	4.6 (2–10)	0.002
Clinical characteristic				
Periodicity	27	40	62	0.05
Oral aphthosis	9.1	35.6	62.5	0.001
Erythematosus pharyngitis	54.5	65	82.8	NS
Exudative pharyngitis	45.4	50	59.3	NS
Enlargement of cervical lymph nodes	72.7	65	85.9	NS
Pain at cervical lymph nodes	18	45	51	NS
Rash	45.4	20	28.1	NS
Conjunctivitis	9	10	17	NS
Periorbital edema	36.3	0	4.6	0.003
Thoracic pain	27.2	5	3.1	0.04
Abdominal pain	81.8	40	50	NS
Diarrhea	18.1	15	26.5	NS
Vomiting	27.2	30	15.6	NS
Splenomegaly	0	25	14	NS
Arthritis	27.2	0	6.2	NS
Arthralgia	63.6	40	43.7	NS
Myalgia	63.6	35	43.7	NS
Headache	54.5	30	31.2	NS

* Except where indicated otherwise, values are the percent of patients. TRAPS = tumor necrosis factor receptor–associated periodic syndrome; PFAPA = periodic fever, aphthosis, pharyngitis, and adenitis syndrome; NS = not significant.

† Determined by Kruskal-Wallis test for heterogeneity.

**Figure 1:**
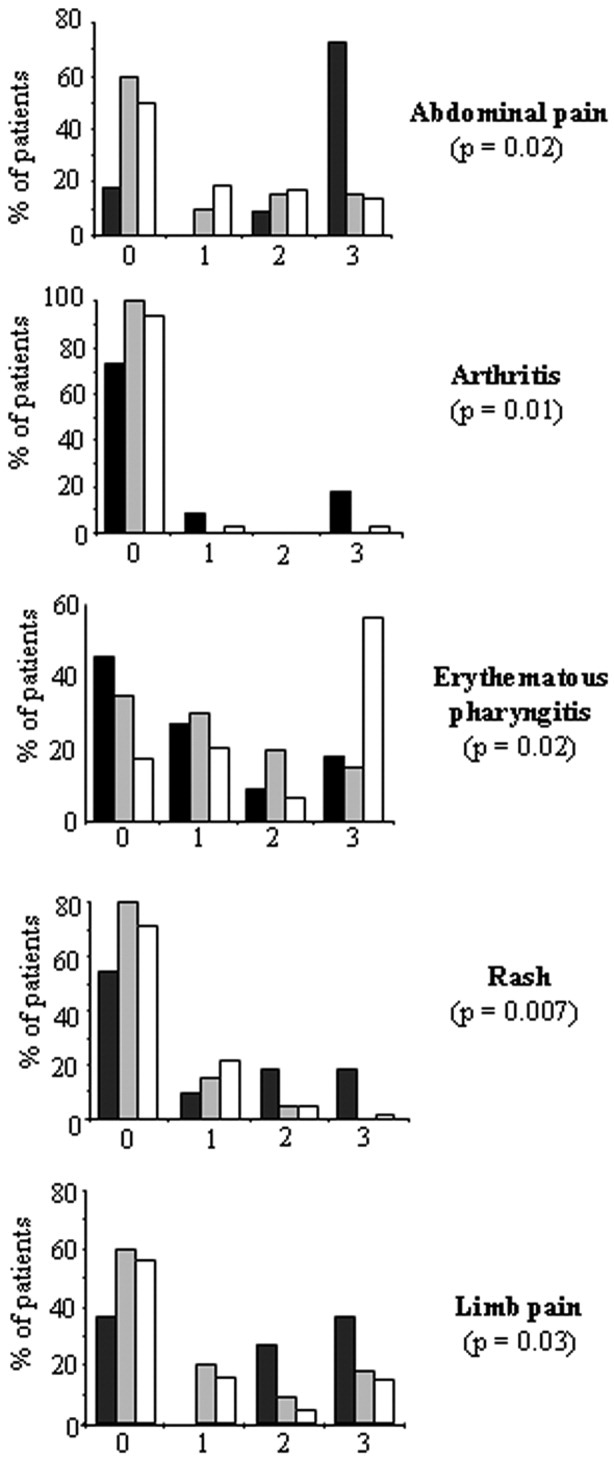
Frequency of typical clinical manifestations in children with tumor necrosis factor receptor–associated periodic syndrome (n = 11; solid bars), children with the R92Q substitution (n = 20; gray shaded bars), and children with periodic fever, aphthosis, pharyngitis, and adenitis syndrome (n = 64; open bars), according to whether the symptom never occurred (score 0), sometimes occurred (score 1), often occurred (score 2), or always occurred (score 3). *P* values indicate the significance of the differences between groups, according to the chi-square test for heterogeneity.

As shown in Table 2, the Kruskal-Wallis test for heterogeneity revealed an increased prevalence of periorbital edema and thoracic pain in patients carrying *TNFRFS1A* structural mutations, whereas patients with PFAPA had an earlier onset of disease and a higher rate of periodicity of fever episodes and oral aphthosis when compared with the other 2 subgroups (Table 2). Moreover, when the clinical symptoms were compared, taking into account not only their presence/absence but also their frequency (never/sometimes/often/always), an even clearer heterogeneity was observed among the 3 subgroups. Indeed, pediatric patients with TRAPS carrying structural mutations of *TNFRFS1A* displayed a higher frequency of abdominal pain, skin rash, and limb pain (myalgia and/or arthralgia) compared to children with the R92Q mutation and children with PFAPA (Figure 1). Conversely, a higher frequency of either erythematous or exudative pharyngitis was observed in patients with PFAPA and, to a lesser extent, in patients with the R92Q mutation compared with patients with TRAPS (Figure 1).

Median laboratory values at the time of fever episodes were available for 46 patients (8 with TRAPS, 12 with the R92Q mutation, and 26 with PFAPA) (Table 3). A high degree of variability in the main laboratory parameters was observed, especially in the R92Q subgroup (Table 3). Apart from a higher elevation of the C-reactive protein level in patients with TRAPS and patients with the R92Q mutation, no other major differences were detected among the 3 subgroups.

**Table 3:** Laboratory parameters during fever episodes in patients with TRAPS, patients with the R92Q mutation, and patients with PFAPA*

	Patients with TRAPS (n = 8)	Patients with R92Q (n = 12)	Patients with PFAPA (n = 26)
WBCs, × 10^3^/mm^3^	16.7 (11.7–41.5)	11.7 (3.5–39.0)	13.7 (4.3–19.8)
Neutrophils, × 10^3^/mm^3^	10.6 (9.0–35.8)	9.3 (2.7–34.6)	8.6 (2.5–16.3)
Monocytes, × 10^3^/mm^3^	0.57 (0.4–0.8)	0.84 (0.3–1.5)	1.01 (0.4–1.7)
Lymphocytes, × 10^3^/mm^3^	2.28 (1.1–5.0)	3.71 (0.5–8.3)	3.14 (1.4–5.9)
Hemoglobin, gm/liter	10.8 (9.1–12.5)	11 (9.3–14.1)	11.6 (11–12.8)
Platelet count, × 10^3^/mm^3^	527 (270–595)	354 (90–433)	352 (220–545)
ESR, mm/first hour	70 (28–95)	57 (26–79)	41 (9–93)
CRP, mg/dl	13.2 (1.3–29)†	11.1 (2–37)	6.1 (2–13.2)

* Values are the median (range). WBCs = white blood cells; ESR = erythrocyte sedimentation rate; CRP = C-reactive protein.

† *P* < 0.05 versus the other groups, by nonparametric Kruskal-Wallis test for heterogeneity. In post hoc analysis by Mann-Whitney U test, differences between groups were as follows: *P* not significant for patients with tumor necrosis factor receptor–associated periodic syndrome (TRAPS) versus patients with R92Q, *P* = 0.005 for patients with TRAPS versus patients with periodic fever, aphthosis, pharyngitis, and adenitis syndrome (PFAPA), and *P* = 0.02 for patients with R92Q versus patients with PFAPA.

### Impact of different *TNFRSF1A* mutations on HRQOL

**Figure 2:**
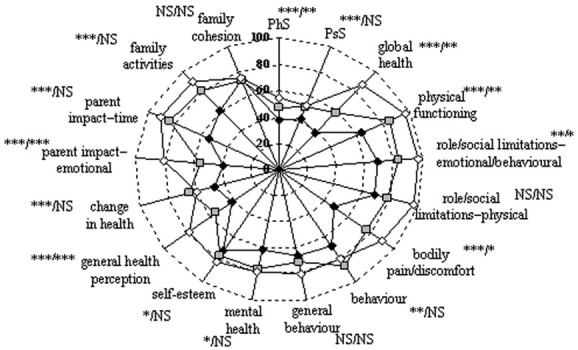
Diagram showing the scores for health-related quality of life (HRQOL) on the 50-item Child Health Questionnaire (score range 0–100) in pediatric patients with tumor necrosis factor receptor–associated periodic syndrome (TRAPS) with structural mutations of the *TNFRSF1A* gene (solid diamonds) and pediatric patients with the R92Q mutation (shaded squares), in comparison with healthy children (open diamonds). HRQOL was assessed as the mean of the 2 summary scores for the physical domain (PhS) and the psychological domain (PsS) and the 15 subscales. Asterisks for each item indicate the *P* values for significant or nonsignificant (NS) differences in HRQOL between TRAPS patients/patients with R92Q as compared with healthy controls. ∗ = *P* < 0.05; ∗∗ = *P* < 0.01; ∗∗∗ = *P* < 0.001, by Mann-Whitney U test.

As shown in Figure 2, scores on the 15 subscales of the CHQ-PF50 and the 2 summary scores (PhS and PsS) were determined at the time of molecular analysis for 9 pediatric patients with TRAPS and 14 pediatric patients with the R92Q mutation, as compared with reference values for healthy controls. *TNFRSF1A* structural mutations were associated with a severe impairment in both the physical domain (PhS score) and the psychosocial domain (PsS score) when compared to the scores in the healthy controls (Figure 2).

Structural mutations had a major impact on most of the items described to assess the HRQOL. The most reduced scores among the CHQ-PF50 health concepts were those related to physical domains, such as global health, physical functioning, and bodily pain/discomfort. However, several items related to psychosocial domains, such as general health perception, emotional impact on parents, family activities, and time impact on parents were also variably impaired (Figure 2). The significantly lower values with regard to health perception, as compared with the values from the preceding year, were consistent with the tendency toward worsening of the disease, as was observed in these patients. Conversely, the impact of disease activity was less severe in patients carrying the R92Q mutation. Notably, the most affected concepts, in comparison with healthy controls, were those associated with global health perception (global health, general health perception, and emotional impact on parents).

### Difference in spontaneous disease course in children with the R92Q mutation compared with children with TRAPS

Data on long-term followup were available for 11 children with TRAPS, 16 children carrying the R92Q mutation, and 64 children with PFAPA. At the time of the study, the mean followup from the time of disease onset was 7.9 years (range 1.6–15 years) for TRAPS patients, 7.3 years (range 1.7–14.3 years) for children with the R92Q substitution, and 5.2 years (range 1.2–13.1 years) for patients with PFAPA. All patients had at least 1 year of followup after the molecular screening.

**Figure 3:**
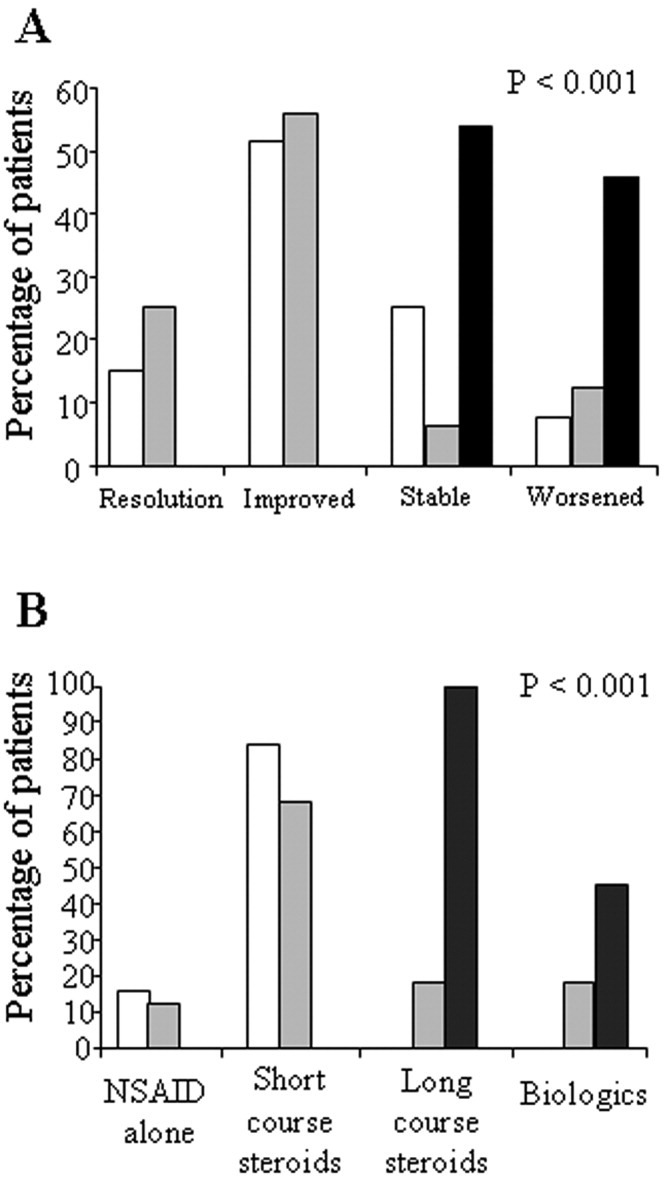
Course of spontaneous disease, defined according to categories of spontaneous resolution, spontaneous improvement, stable disease, or worsened disease (see Patients and Methods), before any continuous treatment **(A)**, and therapeutic strategy at the time of the last followup **(B)** in the 3 subgroups of pediatric patients. The mean followup from disease onset was 7.9 years (range 1.6–15 years) for patients with tumor necrosis factor receptor–associated periodic syndrome (TRAPS) (n = 11; solid bars), 7.3 years (range 1.7–14.3 years) for children with the R92Q substitution (n = 16; shaded bars), and 5.2 years (range 1.2–13.1 years) for children with periodic fever, aphthosis, pharyngitis, and adenitis syndrome (n = 64; open bars). *P* values indicate the significance of the differences in the TRAPS patients compared with the other groups, according to the chi-square test for heterogeneity. NSAID = nonsteroidal antiinflammatory drug.

The spontaneous disease course in the absence of any continuous steroid and/or second-line treatment was evaluated in all patients (Figure 3A). A spontaneous resolution of fever episodes was observed in 4 (25%) of 16 patients with the R92Q mutation, including the patient carrying the mutation in a homozygous state, and in 10 (16%) of 64 patients with PFAPA (Figure 3A). A reduction of more than 30% in the frequency of the fever episodes from baseline was observed in 9 (56%) of 16 patients with the R92Q mutation and 33 (52%) of 64 patients with PFAPA (Figure 3A). Conversely, no spontaneous resolution or reduction of the fever episodes was observed in patients with *TNFRSF1A* structural mutations (Figure 3A). One (6%) of the 16 patients with the R92Q mutation (age at followup 16 years, disease duration 9 years) and 16 (25%) of the 64 patients with PFAFA displayed a persistence of fever episodes without a significant decrease in the frequency of these episodes when compared with that at baseline.

An increased frequency of fever episodes was observed in 5 patients with PFAPA (8%) (Figure 3A). Two patients with the R92Q mutation (13%) showed a worsening of the disease, due to the development of a chronic course that required continuous second-line treatment. The ages at followup of these 2 patients were 16.3 years and 19.8 years, respectively, and the disease durations were 12.3 years and 16.8 years, respectively. The retrospective analysis of the clinical manifestations at the time of disease onset in the 3 patients with R92Q mutations who displayed a stable or progressive disease course did not reveal substantial differences when compared with the other patients carrying the same variant (Table 2). During followup, all patients with structural *TNFRSF1A* mutations displayed a significantly more severe disease course, with persistence of stable disease in 6 patients (54%) and an increase in the frequency or duration of fever episodes in 5 patients (46%) (Figure 3A).

### Therapeutic strategy during followup

The therapeutic strategy used in the different subgroups during followup was then investigated (Figure 3B). Due to the long duration of fever episodes, all TRAPS patients with structural mutations were treated with prolonged courses of steroids (more than 5 days). Due to high cumulative steroid doses, 5 of these patients were treated with second-line biologic therapy (Figure 3B). Recombinant interleukin-1 receptor antagonist (anakinra) was used as a first-choice therapy in 4 children with TRAPS, resulting in a good and persistent response (18). One 12-year-old girl was initially treated with etanercept and showed a good initial response; however, after 1 year, etanercept was withdrawn due to the occurrence of new disease flares. The introduction of anakinra in this patient has led to effective and persistent control of both her clinical symptoms and laboratory results over a followup period of 3 years.

The therapeutic strategy chosen for patients carrying the R92Q variant was largely different from that chosen for patients with TRAPS. Indeed, 11 (68%) of 16 patients with the R92Q mutation were successfully treated with short-term steroids on demand (Figure 3B). Most of these patients displayed a complete response after a single dose of steroids. In 2 patients, treatment with nonsteroidal antiinflammatory drugs (NSAIDs) resulted in control of the clinical manifestations. Only 3 patients (18%) with the R92Q mutation required a prolonged period of steroid treatment to control their clinical and laboratory manifestations. In 1 of these patients, treatment with colchicine was able to significantly reduce the frequency and intensity of fever episodes. The 2 patients in whom a chronic disease course developed became steroid-dependent and were treated with biologic agents. One patient was treated with anakinra, and the disease showed a persistent and complete response after 1 month of treatment (18). The other patient did not respond to treatment with etanercept and displayed a partial response to anakinra, characterized by the absence of clinical manifestations and a slow tapering of the steroid dose, but with a persistent slight elevation in the levels of acute-phase reactants, including the level of serum amyloid A.

Short-term treatment with steroids was the treatment of choice in the majority of patients with PFAPA (54 [84%] of 64) (Figure 3B). All patients displayed prompt control of the clinical manifestations. Other patients were treated exclusively with NSAIDs on demand. Due to the persistence of frequent fever episodes, 6 patients were treated with tonsillectomy, resulting in a good response.

## DISCUSSION

In the present study, we compared patients with recurrent fever according to subgroups of patients carrying the R92Q variation, patients carrying structural mutations in *TNFRSF1A*, and mutation-negative patients with PFAPA. In our study, children with the R92Q mutation displayed a higher degree of similarity with the PFAPA subgroup than with the TRAPS patients carrying structural mutations. This was true for many clinical features, such as the prevalence of a positive family history of recurrent fever and the duration and frequency of fever episodes. We also showed that the incidence and the frequency of the most typical TRAPS-related clinical manifestations (chest pain, abdominal pain, rash, limb pain, and periorbital edema) in patients with the R92Q variant were much more similar to those observed in patients with PFAPA than those observed in patients with structural *TNFRSF1A* mutations. The less severe effect of the R92Q mutations was also confirmed by the milder impact of the disease on many aspects of HRQOL, in contrast to the much more severely affected patients with structural *TNFRSF1A* mutations.

Together with the incidence and frequency of the fever-associated clinical manifestations at the time of the molecular analysis (6,19), the longitudinal observational data on the patients during their long-term followup are an important indicator of the actual impact of the different *TNFRSF1A* mutations on the disease course. This is particularly true for children with periodic fever, especially the mutation-negative patients, who experience a relevant rate of spontaneous resolution of the fever episodes over the long term (12). For this reason, we compared the long-term clinical course of pediatric patients with the R92Q mutation with that of pediatric patients with TRAPS and pediatric patients with PFAPA.

Fortunately, none of our patients developed any of the most typical long-term complications, such as renal amyloidosis, considered to be a major marker of long-term disease severity in adult patients (3–5,19). However, in the present study, we observed that most of the patients with an R92Q mutation experienced a rate of spontaneous resolution or amelioration of fever episodes similar to that observed in mutation-negative patients with a PFAPA phenotype. In this context, the slightly higher percentage of patients with the R92Q mutation experiencing complete resolution, as compared with patients with PFAPA, could be related to a longer followup in the former group. The relatively benign disease course observed in patients with the R92Q mutation was also consistent with the therapeutic strategy used in these patients, generally similar to that reported in patients with PFAPA, i.e., short courses of steroids on demand that were usually effective after single-dose administration (20).

In addition to the low frequency of a positive family history of recurrent fever observed in the majority of the parents of R92Q-positive patients, the results of our followup study add further proof to support the notion of the actual limited pathogenic relevance of this variant in the context of periodic fevers. However, as was also observed in the present study, a few patients carrying the same mutation may present with a more severe disease course (9,19), requiring an aggressive therapeutic approach. This observation has raised the hypothesis of a possible proinflammatory effect of the R92Q mutation that, concomitant with other environmental and genetic factors, may contribute to the inflammatory phenotype observed in these patients. It was also suggested that R92Q mutations could act as a possible susceptibility factor in other autoinflammatory diseases (21) or chronic inflammatory conditions, such as Behçet's disease (22), multiple sclerosis (23), and juvenile idiopathic arthritis (24).

Whether these latter observations simply reflect the relatively high prevalence of the R92Q mutation in the normal population is still a matter of debate (8,25). Indeed, we recently reported that the allele frequency of R92Q in 265 children with periodic fever (2.45%) did not differ significantly from the allele frequency in healthy subjects from the same Italian population (2.25%) (6). The same observation was also reported in a parallel Dutch study (26), supporting the hypothesis that the R92Q mutation should be considered more as a polymorphism than as a pathogenic variant.

The mild impact of the R92Q mutation has also been confirmed by a number of ex vivo and in vitro observations in studies aimed at identifying the functional impact of different mutations of *TNFRSF1A*. Structural mutations of *TNFRSF1A* have been associated with the following pathogenic consequences: 1) defective shedding of TNFRI from cell membranes after cell activation (3,6), 2) impaired TNF-induced apoptosis in fibroblasts and neutrophils (6,27), and 3) formation of intracellular aggregates of mutated TNFRI that cause impaired trafficking of the receptor to the cell membrane (28,29). Recent evidence supports the hypothesis that intracellular accumulation of the mutated receptor causes a ligand-independent activation of kinases, leading to aberrant cytokine production (30). Notably, in all of the above-mentioned studies, the behavior of the R92Q variant of TNFRI was much more similar to that of the wild-type receptor than to that of the structural *TNFRSF1A* mutations (3,6,28,29).

These observations, in accordance with the genetic and clinical findings reported above, seem to represent a reappraisal of the actual pathogenic role of this variant. In our study, we provide evidence that patients with the R92Q mutation do not present the same clinical picture and outcomes as those observed in patients with TRAPS, and that patients with the R92Q mutation do have similarities with many other mutation-negative patients with periodic fever.

Despite the relatively small number of patients with *TNFRSF1A* mutations analyzed in the present study, our decision to screen all 3 genes and to longitudinally observe the patients allowed us to verify the actual incidence and clinical impact of the R92Q mutation in a homogeneous population of children with periodic fever. International registries on these rare diseases, such as Eurofever (available at http://www.printo.it/eurofever), will facilitate verification of these observations in a larger number of patients from different countries and of different ethnicities, which might conclusively change the current thinking about the clinical significance of the R92Q mutation.

In summary, our study shows that the majority of children with the R92Q mutation of *TNFRSFA1* display a milder disease course as compared with patients with structural mutations and have a higher rate of spontaneous resolution and amelioration of episodic fever. This clinical observation supports the notion of the limited pathogenic role of this variant. Based on these data and on the prevalence of this variant in the normal population, great caution should be taken in the interpretation of positive findings for the R92Q variant in molecular analysis of children with periodic fever, in either epidemiologic studies or clinical studies.
